# A PILOT STUDY OF YIN YOGA AND THE EFFECTS ON BURDEN AND SELF-COMPASSION IN CAREGIVERS OF PERSONS WITH DEMENTIA

**DOI:** 10.1093/geroni/igad104.2971

**Published:** 2023-12-21

**Authors:** Suzanne E Parkman, Jill Olausson

**Affiliations:** University of Southern Maine, Portland, Maine, United States; University of Southern Maine, Portland, Maine, United States

## Abstract

Informal caregivers for persons with dementia face tremendous challenges in balancing their own needs and those of the care recipient. The purpose of this pilot study was to test the feasibility and acceptability of a Yin yoga intervention on caregiver burden and self-compassion. Authors explored recruitment, retention, adherence, and satisfaction with the yoga intervention. Using the three elements of the Neff’s Model of Self-compassion, this pilot study was a quasi-experimental pre/post comparisons of caregiver burden (Zarit Burden Interview Scale-ZBI) and self-compassion (Neff Self-compassion Scale-SCS) in which the participants served as their own control group. A total of five participants (N=5) were recruited and completed an 8-week Yin yoga intervention via Zoom. Statistical analysis revealed non-significant results for ZBI (t (4) = .95, p= .39) and SCS (t(4) = -.23, p= .83). Wilcoxon signed rank test identified two questions on ZBI pre-and post-intervention had differences approaching significance (p= .059). Despite the non-significant results, burden was decreased in three participants and self-compassion scores were increased in four participants. The main perceived benefits were ease of survey process, feeling relaxed after intervention, and treatment fidelity. The main barriers identified were recruitment and retention of participants throughout the 8-week intervention. Overall, we found the study protocol to be feasible and acceptable. Possible modifications to the study would include extending the scope of the study nationally in efforts to recruit maximum participants. Future research is needed with larger sample sizes, randomization, control of confounding variables, and efforts to promote recruitment and retention of participants.

